# The relationship between abdominal pain and emotional wellbeing in children and adolescents in the Raine Study

**DOI:** 10.1038/s41598-020-58543-0

**Published:** 2020-02-03

**Authors:** Oyekoya T. Ayonrinde, Oyedeji A. Ayonrinde, Leon A. Adams, Frank M. Sanfilippo, Therese A. O’ Sullivan, Monique Robinson, Wendy H. Oddy, John K. Olynyk

**Affiliations:** 1 Department of Gastroenterology and Hepatology, Fiona Stanley Hospital, Murdoch, WA Australia; 2 Medical School, The University of Western Australia, Perth, WA Australia; 3 Faculty of Health Sciences, Curtin University, Bentley, WA Australia; 4 Departments of Psychiatry and Psychology, Queens University, Kingston, ON Canada; 5 Providence Care Hospital, Kingston, ON Canada; 6 Department of Hepatology, Sir Charles Gairdner Hospital, Nedlands, WA Australia; 7 School of Population and Global Health, The University of Western Australia, Perth, WA Australia; 8 Department of Pharmacy, Royal Perth Hospital, Perth, Western Australia; 9 School of Medical and Health Sciences, Edith Cowan University, Joondalup, WA Australia; 10 Telethon Kids Institute, The University of Western Australia, Nedlands, WA Australia; 11 Menzies Institute for Medical Research, University of Tasmania, Hobart, TAS Australia

**Keywords:** Gastroenterology, Signs and symptoms

## Abstract

Abdominal pain is a common reason for medical visits. We examined the prevalence, gastrointestinal, and emotional significance of abdominal pain in a population-based cohort serially followed up from birth to 17 years. Children and adolescents from Generation 2 of the Raine Study participated in comprehensive cross-sectional assessments at ages 2, 5, 8, 10, 14 and 17 years. At 17 years, medical history, general health, gastrointestinal symptoms, medications, health practitioner attendance, and self-rated unhappiness were recorded. Longitudinal data regarding abdominal pain or unhappiness, from serial questionnaires, were analysed to identify factors associated with abdominal pain and adverse emotional health at age 17 years. Females experienced more abdominal pain than males at all ages (p < 0.05). Seventeen-year-old adolescents with abdominal pain reported a higher prevalence of depression, anxiety, being bullied at school, and poorer health status than those without abdominal pain (p < 0.05 for all). Abdominal pain and unhappiness during childhood and mid-adolescence were prospectively associated with recurrent abdominal pain, anxiety, depression and unhappiness during late adolescence (p < 0.05 for all). In conclusion, abdominal pain in children and adolescents associates with depression, anxiety, being bullied, unhappiness and reduced overall health-rating during adolescence. Awareness of these factors may guide management decisions.

## Introduction

Abdominal pain is one of the most common reasons for primary care, hospital outpatients or emergency department visits, hospitalisations, referrals to gastroenterologists and for imaging tests^1–7^. In 2016/2017, abdominal and pelvic pain was the most common principal diagnosis for emergency department presentations and hospitalisations in Australia, accounting for 4.3% of principal diagnoses^8^. Likewise, in the USA in 2014/2015, abdominal pain was the most frequent reason for gastrointestinal-related ambulatory medical visits to primary care and emergency departments, with an expenditure of US$10.2 billion^2^. In Australia, abdominal pain accounted for 2.1 per 100 patient primary care visits, and the aetiology was not confirmed in one third of males and half of females^9^. Only one third of patients were prescribed medications, primarily paracetamol or hyoscine butylbromide for the pain, while counselling was the most common non-pharmacological treatment. This suggests a suspected diagnosis of a functional abdominal pain disorder^10^. Rome IV diagnostic criteria for functional gastrointestinal disorders describe four main categories of functional abdominal pain disorders in children and adolescents. These comprise functional dyspepsia, irritable bowel syndrome, abdominal migraine and functional abdominal pain - not otherwise specified, which can coexist with each other and with other medical conditions associating with gastrointestinal symptoms in the same individual^11^.

Amongst Australian adolescents, females present to general practitioners with abdominal pain more frequently than males^9,12^. In adolescents in the Raine Study we recently described sex-specific patterns of gastrointestinal symptoms that were associated with diet, emotional health, health practitioner attendance, and adverse everyday wellbeing^13^. Also, in a large German survey 36% of children and 29% of adolescents reported recurrent abdominal pain over a 3-month period, with a higher prevalence in girls^14^. An association between childhood depressive symptoms and subsequent functional abdominal pain during adolescence has been described in a longitudinal study in the USA^15^. Chronic abdominal pain in adolescents is commonly associated with other somatic pain, interference with education, employment and quality of life^16^, and adversely affects mental health^17^. Consequently, abdominal pain present or persisting during adolescence risks progressing into chronic abdominal pain that adversely affects various facets of life, including relationships, enjoyment of food, and social and workforce participation during adulthood. Chronic abdominal pain may result in attendance with various health practitioners to address abdominal pain, other somatic pain, depression or anxiety, dietary interventions, or self-medication with prescription, over the counter medications or other substances. Consequently, chronic or persisting abdominal pain in adolescents may be negatively associated with physical and emotional wellbeing and is deserving of attention.

## Aims

The aims of the current study were to describe (a) patterns of abdominal pain and the relationship between abdominal pain, other gastrointestinal symptoms and medication use in adolescents, (b) the prospective relationship between abdominal pain and unhappiness experienced during childhood and adolescence, and abdominal pain, depression, anxiety or unhappiness during late adolescence, and (c) to identify risk factors for frequent abdominal pain in a cohort of population-based adolescents.

## Methods

This is an observational study examining gastrointestinal and emotional health correlates of abdominal pain in children and adolescents participating in the Raine Study.

### The Raine Study

The Raine Study is a predominantly-Caucasian cohort study established between 1989–1991 as a randomised controlled trial evaluating the effects of repeated antenatal ultrasound on the course of pregnancy and pregnancy outcomes^18^. At the outset, the study involved serial assessments of 2868 women (Generation 1 or Gen1) at approximately 18 weeks into pregnancy. The offspring (Generation 2 or Gen2) have been prospectively followed from birth into adulthood^19^. References to children and adolescents in the analyses in this study relate to Gen2 Raine Study participants. Institutional ethics committee approval was obtained from the Princess Margaret Hospital for Children Human Research Ethics Committee. Signed informed consent from parents or legal guardians was obtained for each child and adolescent assessment, and adolescent assent at age 17 years was obtained. All research was performed in accordance with the approved guidelines.

#### Longitudinal assessments

During serial Raine Study follow up assessments at ages 2, 8 and 10 years the parent recorded the presence of abdominal pain related to bowel motions, while at age 17 years the Gen 2 adolescent reported the presence of abdominal pain. Abdominal pain was reported: (a) by frequency at ages 2, 8, 10 and 17 years, (b) as being present or absent with bowel motions at ages 2 and 10 years, and (c) severe enough to interfere with usual activities on at least three episodes during the preceding three months in eight-year-olds. Characteristics of the abdominal pain and associated gastrointestinal symptoms in children were described by the parent at different ages and by the adolescent at age 17 years. The primary care physician diagnosis for the abdominal pain was reported at age 10 years. Questionnaire data regarding abdominal pain was available for 1896, 2108, 2021 and 1281 children or adolescents who participated in the 2, 8, 10 or 17-year cross-sectional assessments, respectively, of the Raine Study. Whether the child or adolescent felt generally unhappy or sad or not was documented on an ordinal scale from ‘not true’ to ‘sometimes true’ to ‘often true’ by the parent for children aged 2–10 years, and subsequently by the adolescent during follow up at ages 14 and 17 years.

#### Seventeen-year cross-sectional assessment

Seventeen-year-old Gen2 adolescents in the Raine Study participated in a cross-sectional assessment that involved physical assessments and detailed self-administered questionnaires regarding health and lifestyle. Questions included medical history, incorporating gastrointestinal symptoms, diagnoses, health professional attendance and medications used. The questions asked about the presence and frequency of abdominal pain (excluding menstrual period pain) and associated gastrointestinal symptoms during the preceding 3 months. Use of classes of medications, including analgesics, laxatives, anti-diarrhoeal, and antidepressant medications were recorded. The consistency of stool was reported as ‘very hard’, ‘hard’, ‘not too hard and not too soft’, ‘very soft or mushy’, or ‘watery’. Health professional consultation and health professional-diagnosed medical conditions, including a past and/or current diagnosis of depression or anxiety during the preceding 12 months were also documented. Adolescents reported nausea and vomiting as absent, sometimes, or often, and rated their overall health status on a scale using the descriptors “poor”, “fair”, “good”, “very good” or “excellent”. We defined nausea or vomiting reported as often experienced as frequent nausea or frequent vomiting. The parent or primary care provider independently completed a questionnaire regarding the health and lifestyle of the adolescent, and this was used to verify medications, diagnoses and health professional attendance. The parent or primary care provider will henceforth be referred to as parent in this study. In this study, the term adolescent refers to the 17-year-old unless age 14 years is specified. Body mass index was calculated as weight (kg)/height(m)^2^.

### Statistical analysis

Continuous descriptive data were summarised as means and standard deviations, with categorical variables as proportions. The main outcome variables were the presence and frequency of abdominal pain, report of being unhappy, and the self-rated health status. Differences in continuous variables were compared using the independent t test. Differences between categorical variables were determined with the Pearson chi-square test or Fisher’s exact test. All p values were reported as two-sided and were interpreted at the 5% level of significance. Multivariable logistic regression models were used to calculate the odds of abdominal pain, depression, anxiety or self-rated poor-severe health status from abdominal pain, other gastrointestinal symptoms, depression, anxiety, reported unhappiness at different childhood and adolescent ages, and medications that were statistically significant in univariate analysis. Interaction terms were tested between abdominal pain and depression, and between depression and sex but no significant interaction was identified. Data were analyzed using IBM SPSS statistics for Windows (version 20.0; IBM Corp., Armonk, NY).

## Results

### Abdominal pain

#### Age 17 years

The three-month prevalence of abdominal pain was 457/1281 (35.7%), including at least once per week in 7% of adolescents. The usual stool frequency and consistency patterns in adolescents with or without abdominal pain are summarized in Table 1. Those with abdominal pain experienced more abdominal bloating, hard stool, nausea and vomiting than those without abdominal pain and were more commonly female (Table 1). The duration of abdominal pain was three hours or longer per episode in 117 (25.7%) and improved after having a bowel motion in 352 (77.9%). When abdominal pain was experienced ≥1 day per week (n = 89), there was an associated change in frequency or consistency of bowel motions in 72/89 (80.9%). Abdominal pain ≥1 day per week in adolescents was associated with bloating ≥1 day per week (unadjusted OR 5.96, 95% CI 3.04–11.73, p < 0.001). There was no difference in BMI between those with abdominal pain and those without abdominal pain. Amongst the adolescents, there was no prevalent diagnosis of inflammatory bowel disease.

**Table 1:** Characteristics of 17-year-old adolescents in the Raine cohort based on the presence or absence of abdominal pain.

	Total (n = 1281)	Abdominal pain	No abdominal pain	P value
Sex				
• Female	676/1281 (52.8%)	281/676 (41.6%)	395/676 (58.4%)	<0.001
• Male	605/1281 (47.2%)	176/605 (29.1%)	429/605 (70.9%)	
Frequency of bowel motions				
• 0–2 per week	189 (14.8%)	71 (15.6%)	118 (14.4%)	0.1
• 3–6 per week	457 (35.9%)	180 (40.0%)	275 (33.6%)	
• 1 per day	489 (38.4%)	154 (33.8%)	335 (41.0%)	
• 2–3 per day	131 (10.3%)	45 (9.9%)	86 (10.5%)	
• >3 times per day	7 (0.5%)	3 (0.7%)	4 (0.5%)	
Self-reported stool characteristics and gastrointestinal symptoms				
• Stool not too hard and not too soft	1140/1267 (90%)	393/453 (86.8%)	717/814 (91.8%)	0.04
• Stool soft, mushy or watery	40/1267 (3.2%)	17/453 (3.8%)	23/814 (2.8%)	0.37
• Stool hard or very hard	87/1276 (6.9%)	43/453 (9.5%)	44/814 (5.4%)	0.006
• Abdominal bloating	915/1275 (71.8%)	397/456 (87.1%)	518/819 (63.2%)	<0.001
• Nausea	262/1057 (24.8%)	144/378 (38.1%)	118/669 (17.4%)	<0.001
• Vomiting	94/1053 (8.9%)	58/375 (15.5%)	36/678 (5.3%)	<0.001
Depression	72/1157 (6.2%)	40/405 (9.9%)	32/752 (4.3%)	<0.001
Anxiety	119/1164 (10.2%)	58/409 (14.2%)	61/755 (8.1%)	0.001
Self-reported poor-fair health rating	132/1280 (10.3%)	68/457 (14.9%)	64/823 (7.8%)	<0.001
Body mass index (kg/m^2^)	23.0 (5.8)	23.2 (7.1)	22.9 (5.0)	0.51

Footnote: Adolescent sex, self-reported frequency and consistency of bowel motions, gastrointestinal symptoms, health rating and medically-diagnosed depression and anxiety are presented as proportions, while body mass index is presented as mean (standard deviation). P values are for comparisons between adolescents with abdominal pain and adolescents without abdominal pain using Chi square test or Student’s t test. P < 0.05 is considered statistically significant.

#### Age 10 years

At age 10 years, 111/2014 (5.5%) children had abdominal pain during at least 12 weeks of the preceding year (6.7% girls vs. 4.4% boys, p = 0.02). Abdominal pain was predominantly experienced in the lower abdomen (49.8%) vs. peri-umbilical (37.2%) vs. upper abdomen (13.0%). There were no sex differences in the location of the abdominal pain. Further, abdominal pain was associated with painful bowel motions in 159/1999 (8.0%), improved with bowel motions in 457/1963 (23.3%), was associated with a change in stool consistency in 231/1916 (12.1%) and with a change in stool frequency in 234/1932 (12.1%). The prevalence of severe abdominal pain increased with the presence of constipation (none or mild abdominal pain 12.1% vs. severe abdominal pain 29.1%, p < 0.001). Similarly, the prevalence of painful bowel motions increased with the frequency of constipation (no constipation 4.3% vs. sometimes/ often constipated 40.9%, p < 0.001). Children with abdominal pain at 10 years were more likely to experience abdominal pain ≥1 day per week at age 17 years (unadjusted OR 5.17, 95% CI 2.75–9.73, p < 0.001) (Table 2). Abdominal pain described as sometimes or always severe in 10-year-olds, was associated with increased likelihood of abdominal pain occurring on ≥1 day per week at age 17 years (unadjusted OR 2.25, 95% CI 1.32–3.84, p = 0.003). Also, lower abdominal pain, compared with central plus upper abdominal pain, at age 10 years was associated with abdominal pain at age 17 years (unadjusted OR 1.49, 95% CI 1.04–2.13, p = 0.03).

**Table 2:** Relationship between abdominal pain during childhood and recurrent abdominal pain in 17-year-old adolescents in the Raine Study.

Age (years)	Characteristic of abdominal pain	Abdominal pain ≥ 1 day per week at age 17 years					
		Univariate analysis			Multivariable logistic regression analysis		
		OR	95% CI	P value	OR	95% CI	P value
2	Painful bowel motions	2.36	1.28–4.32	0.006			
8	>3 episodes interfering with usual activities in past 3 months	2.32	1.21–4.48	0.01			
	Daily to several times per month	4.85	2.28–10.30	<0.001	7.33	1.50–35.87	0.01
10	Frequency ≥12 weeks of the preceding year	5.17	2.75–9.73	<0.001	4.12	2.09–8.12	<0.001
	Painful bowel motions	3.6	1.98–6.55	<0.001	2.4	1.18–4.88	0.02
	Improves with bowel motion	2.19	1.36–2.52	0.001	1.43	0.82–2.52	0.21
	Pain diagnosis						
	Psychological	1.13	0.15–8.79	0.91			
	Constipation	2.12	0.80–5.58	0.13			
	Abdominal migraine	1.94	0.24–15.99	0.54			

Abdominal pain characteristics were reported by the parent for children and self-reported by the adolescent. Data are presented as odds ratios and 95% confidence intervals (CIs). P values are for associations between abdominal pain characteristics during childhood and abdominal pain during adolescence using logistic regression analysis. P < 0.05 is considered statistically significant.

#### Age 8 years

At age 8 years, 142/2108 (6.7%) children had ≥3 episodes of abdominal pain severe enough to interfere with their usual activities during the preceding three months. The frequency of abdominal pain increased with the presence of constipation (no constipation 1.8% vs. sometimes/ often constipated 11.7%, p < 0.001). Abdominal pain was more common in girls than boys (8.5% vs. 5.1%, p = 0.002) and was associated with subsequent abdominal pain at age 10 years (unadjusted OR 9.53, 95% CI 5.32–17.10, p < 0.001), and with abdominal pain ≥1 day per week at age 17 years (unadjusted OR 2.32, 95% CI 1.21–4.48, p = 0.01). (Table 2).

#### Age 2 years

At age 2 years, 193/1896 (10.2%) of children had painful bowel motions, according to the parent (11.7% girls vs. 8.7% boys, p = 0.03). The prevalence of painful bowel motions increased with the presence of constipation (no constipation 4.6% vs. sometimes/ often constipated 59.4%, p < 0.001). Painful bowel motions at age 2 years were associated with subsequent abdominal pain at age 8 years (unadjusted OR 3.67, 95% CI 2.06–6.51, p < 0.001) and abdominal pain ≥1 day per week at age 17 years (unadjusted OR 2.36, 95% CI 1.28–4.32, p = 0.006) (Table 2).

### Emotional wellbeing - Depression, anxiety, bullying and self-reported unhappiness

#### Depression and anxiety at age 17 years

A past and/or current diagnosis of depression was reported in 6.2% (8.6% female vs. 3.7% male, p = 0.001) and a diagnosis of anxiety in 10.2% (12.7% female vs. 7.6% male, p = 0.001) of 17-year old adolescents. Children and adolescents reporting abdominal pain had a higher prevalence of depression and anxiety at age 17 years, compared with those without abdominal pain (Tables 3 and 4). In particular, adolescents with abdominal pain on one or more days per week were three times more likely to have a diagnosis of depression than other adolescents.

**Table 3:** Relationship between abdominal pain during childhood and adolescence and depression in 17-year-old adolescents in the Raine Study.

Age (years)	Characteristic of abdominal pain	Depression at age 17 years					
		Univariate analysis			Multivariable logistic regression analysis		
		OR	95% CI	P value	OR	95% CI	P value
2	Painful bowel motions	0.78	0.30–2.00	0.6			
8	≥3 episodes interfering with usual activities in past 3 months	2.19	1.04–4.60	0.04	1.44	0.26–7.98	0.67
	Frequency several days per week	2.88	0.63–13.26	0.18			
10	Frequency ≥12 weeks of the preceding year	2.15	1.12–4.12	0.02	1.99	0.78–5.08	0.15
	Painful bowel motions	1.57	0.83–2.98	0.17			
	Improves with bowel motion	1.56	1.02–2.38	0.04			
	Pain diagnosis						
	Psychological	4.76	1.44–15.72	0.01	4.14	1.22–14.03	0.02
	Constipation	3.2	1.48–6.90	0.003	2.98	1.37–6.51	0.006
	Abdominal migraine	0.95	0.12–7.40	0.96			
	Gastro-oesophageal reflux	1.31	0.16–10.53	0.8			
17	Occurrence during preceding 3 months	2.47	1.52–3.99	<0.001			
	Frequency ≥1 day per week during preceding 3 months	3.43	1.75–6.40	<0.001	2.46	1.16–5.21	0.02

Depression was medically-diagnosed, while the abdominal pain characteristics were reported by the parent for children and self-reported by the adolescent. Data are presented as odds ratios and 95% confidence intervals (CIs). P values are for associations between abdominal pain characteristics and depression using logistic regression analysis. P < 0.05 is considered statistically significant.

**Table 4:** Relationship between abdominal pain during childhood and adolescence and anxiety in 17-year-old adolescents in the Raine Study.

Age (years)	Characteristic of abdominal pain	Anxiety at age 17 years					
		Univariate analysis			Multivariable logistic regression analysis		
		OR	95% CI	P value	OR	95% CI	P value
2	Painful bowel motions	0.53	0.23–1.25	0.15			
8	≥3 episodes interfering with usual activities in past 3 months	2.45	1.36–4.41	0.04	3	1.66–5.41	<0.001
	Frequency several days per week	5.82	1.87–18.12	0.002			
10	Frequency ≥12 weeks of the preceding year	3.3	1.70–6.42	<0.001	3.21	1.62–6.35	0.001
	Painful bowel motions	1.92	0.95–3.86	0.07			
	Improves with bowel motion	1.86	1.14–3.01	0.01	1.65	1.01–2.72	0.047
	Pain diagnosis						
	Psychological	2.83	0.62–13.01	0.18			
	Constipation	3.47	1.48–8.12	0.004			
	Abdominal migraine	1.4	0.18–10.97	0.75			
	Gastro-oesophageal reflux	1.93	0.24–15.61	0.54			
17	Occurrence during preceding 3 months	1.88	1.28–2.75	0.001			
	Frequency ≥1 day per week during preceding 3 months	3.78	2.22–6.44	<0.001	2.1	0.56–7.89	0.27

Anxiety was medically-diagnosed, while the abdominal pain characteristics were reported by the parent for children and self-reported by the adolescent. Data are presented as odds ratios (OR) and 95% confidence intervals (CIs). P values are for associations between abdominal pain characteristics and depression using logistic regression analysis. P < 0.05 is considered statistically significant.

#### Victims of bullying at school

In the cohort, 41.7% of adolescents (50.7% with abdominal pain vs. 36.7% without abdominal pain, p < 0.001) reported that they had been bullied at school. Adolescents with recurrent abdominal pain were twice as likely to have been bullied at school. The prevalence of abdominal pain increased with the frequency of being bullied at school; from not bullied to bullied less than twice per month to bullied more than once per week (30.9% to 42.3% to 47.6%, p = 0.006).

#### Parent-reported and self-reported unhappiness

**Figure 1:**
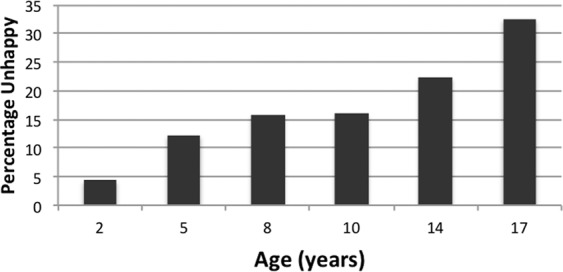
Longitudinal reports of the child or adolescent in Raine Study being unhappy. Reports of child unhappiness at ages 2,5,8 and 10 years were provided by the parent but self-reported by the adolescent at ages 14 and 17 years.

**Figure 2:**
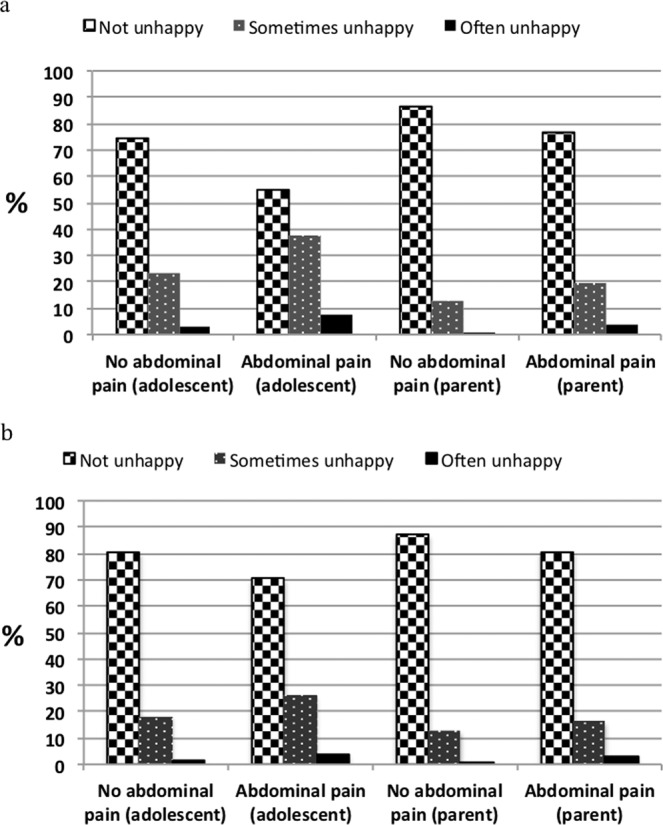
(**a**,**b**) Relationship between abdominal pain and unhappiness in adolescents in the Raine Study at age (**a**) 17 years and (**b**) 14 years. Bars represent unhappiness in the adolescent based on self-report or parent report, respectively. Adolescents with abdominal pain were significantly more unhappy than those not experiencing abdominal pain (p < 0.001).

The prevalence of parent reports of the child being unhappy and subsequent adolescent self-reports of feeling unhappy increased with age from 4.4% at age 2 years to 32.6% at age 17 years (Fig. 1). During childhood, painful bowel motions were associated with corresponding parent reports of the child being unhappy (OR 2.43, 95% CI 1.39–4.24, p = 0.002 at 2 years; OR 1.81, 95% CI 1.23–2.65 at 10 years). Similarly, frequent abdominal pain was associated with parent report of child unhappiness at age 8 years (OR 2.57, 95% CI 1.47–4.48, p = 0.001) and at 10 years (OR 3.07, 95% CI 2.03–4.65, p < 0.001). Reports of the child or adolescent being unhappy at ages 5, 8, 10 and 14 years were significantly associated with a subsequent report of being unhappy, having anxiety, depression, or experiencing abdominal pain as a 17-year-old adolescent (p < 0.05 for all, data not shown). However, being unhappy during childhood or adolescence was not consistently associated with the frequency of abdominal pain during adolescence (Table 5). Abdominal pain was more likely if one parent did not live in the household (OR 1.79, 95% CI 1.01–3.18) or the adolescent felt unhappy (OR 2.38, 95% CI 1.66–3.40), after adjusting for anxiety and depression. Recurrent abdominal pain in adolescents was associated with child reports of being unhappy at age 8 years and teacher reports that the child was unhappy at age 10 years (OR 1.75, 95% CI 1.02–2.99 and OR 2.14, 95% CI 1.21–3.78, respectively). Adolescent self-report of feeling unhappy, and parent report that the adolescent was unhappy when aged 14 and 17 years were associated with abdominal pain at age 17 years (p < 0.001, Fig. 2a,b respectively).

**Table 5:** Relationship between frequent abdominal pain in adolescents, and unhappiness in children and adolescents, and gastrointestinal symptoms, depression and anxiety in 17-year-old adolescents in the Raine Study.

Age (years)	Characteristic	Abdominal pain ≥1 day per week at age 17 years					
		Univariate analysis			Multivariable logistic regression analysis		
		OR	95% CI	P value	OR	95% CI	P value
2	Sex (female)	1.65	1.05–2.58	0.03	1.77	0.65–4.80	0.27
5	Unhappy child	1.29	0.46–3.61	0.62			
8	Unhappy child	1.51	0.79–2.87	0.21			
	Abdominal pain several times per week	5.48	1.68–17.86	0.005	1.52	0.15–15.51	0.73
	Unhappy child	1.75	1.02–3.00	0.04	6.09	1.08–34.04	0.04
10	Abdominal pain ≥12 times per year	5.17	2.75–9.73	<0.001	4.85	1.56–15.06	0.006
	Unhappy child	1.57	0.90–2.74	0.12			
14	Unhappy adolescent	1.61	0.97–2.68	0.07			
17	Abdominal bloating ≥1 day per week	6.54	4.18–10.24	<0.001	4.11	1.99–8.50	<0.001
	Frequent nausea	8.35	4.06–17.20	<0.001	2.24	0.63–7.98	0.21
	Frequent vomiting	10.94	2.87–41.65	<0.001	7.89	0.88–7.94	0.07
	Anxiety	3.78	2.22–6.44	<0.001	1.64	0.58–4.65	0.35
	Depression	3.34	1.75–6.40	<0.001	0.97	0.27–3.49	0.67
	Diarrhoea	1.96	0.44–8.75	0.38			
	Constipation	1.61	0.99–2.64	0.06			
	Unhappy adolescent	3.67	2.25–6.00	<0.001	1.98	0.94–4.18	0.07
	Bullied at school	2	1.11–3.60	0.02	1.42	0.65–3.09	0.38

Depression and anxiety were medically-diagnosed. Abdominal pain characteristics and unhappiness were reported by the parent for children, while gastrointestinal symptoms, unhappiness and a history of being bullied were selfreported by the 14 or 17-year old adolescent. Data are presented as odds ratios (OR) and 95% confidence intervals (CIs). P values are for associations between frequent abdominal pain at age 17 years and preceding abdominal pain, unhappiness or prevalent gastrointestinal symptoms, depression, anxiety and unhappiness using logistic regression analysis. P < 0.05 is considered statistically significant.

### Prediction of abdominal pain

Using multivariable logistic regression analysis, recurrent abdominal pain at age 17 years was associated with reports of being unhappy at age 8 years, recurrent abdominal pain at age 10 years and frequent abdominal bloating at age 17 years after adjusting for sex, diarrhoea, constipation, bullying, anxiety and depression at age 17 years (Table 5).

### Medication use and health practitioner attendance

#### Medication use

Medication use by 17-year-old adolescents in the cohort, and associations between medication use and abdominal pain are summarized in Table 6. Medications most commonly used by adolescents were analgesics (54.1%) and vitamins or minerals (25.6%). There was a higher likelihood of use of analgesics, antidepressants, laxatives, and vitamins and minerals in adolescents with abdominal pain compared with those without abdominal pain (p < 0.05 for all). Adolescents with abdominal pain had a higher prevalence of analgesic medication use (64.9% vs. 48.2%, p < 0.001) and antidepressant medication use (3.6% vs. 0.7%, p = 0.001) compared with adolescents without abdominal pain. Female sex and use of analgesics or antidepressants were independently associated with abdominal pain after adjusting for use of laxatives, anti-emetics, intestinal motility, anti-diarrhoeal medications, or vitamins and minerals (Table 6).

**Table 6:** Association between abdominal pain and medications used by the 17-year-old adolescents during the preceding 3 months.

Medication	Prevalence	Abdominal pain					
		Univariate analysis			Multivariable logistic regression analysis		
		OR	95% CI	P value	OR	95% CI	P value
Sex (female)	52.8%	1.73	1.37–2.19	<0.001	1.59	1.19–2.10	0.001
Analgesics	54.1%	1.99	1.56–2.53	<0.001	2.04	1.53–2.73	<0.001
Antidepressant	1.8%	5.07	1.81–14.91	0.002	3.46	1.13–10.62	0.03
Antidiarrhoeal medications	2.0%	3.59	0.33–39.70	0.30			
Laxatives	2.7%	2.63	1.31–5.25	0.006	1.94	0.86–4.40	0.11
Vitamins and minerals	25.6%	1.41	1.08–1.83	0.01	1.10	0.80–1.51	0.57
Intestinal motility/antispasmodic drugs	0.8%	17.28	1.90–156.83	0.01	3.01	0.52–17.55	0.22
Antiemetics	0.6%	1.39	0.31–6.24	0.67			

Medications were reported by the adolescent and/ or parent. OR = odds ratio (reference is no abdominal pain); CI = confidence intervals. P values are for associations between abdominal pain in adolescents, sex and medication use. P < 0.05 is considered statistically significant. Female sex, use of analgesics or antidepressants were independently associated with abdominal pain in adolescents.

#### Health practitioner attendance

Adolescents with abdominal pain were more likely to have attended a primary care physician, psychologist or psychiatrist, dietitian, or school nurse during the preceding 12 months than those without abdominal pain (p < 0.05 for all).

### Perceived health status

Adolescents with abdominal pain self-reported their health as being mostly, or definitely, not excellent twice as often as those without abdominal pain (23.6% vs. 11.2%, p < 0.001). Frequent abdominal pain was more likely to be associated with “poor” or “fair” health, and less likely to be associated with “very good” or “excellent” health than less frequent abdominal pain. Table 7 summarises the association between adolescent characteristics and poor-fair overall health rating in adolescents with abdominal pain. In univariate analysis, factors associated with increased odds of describing poor-fair health status in adolescents with abdominal pain were depression, nausea, feeling unhappy and anxiety. In multivariate analysis, depression, nausea and feeling unhappy were associated with poorer self-reported health status after adjusting for other variables (Table 7).

**Table 7:** Association between gastrointestinal and mental health characteristics and poor-fair overall health rating in 17-year-old adolescents.

Characteristic	Poor-fair overall health rating					
	Univariate analysis			Multivariable logistic regression analysis		
	OR	95% CI	P value	Adjusted OR	95% CI	P value
History of depression	5.54	2.74–11.18	<0.001	6.10	2.64–14.10	<0.001
Frequent nausea	5.66	2.42–13.28	<0.001	2.42	1.23–4.76	0.01
Unhappy	2.83	1.54–5.19	<0.001	2.02	1.01–4.05	0.048
Anxiety	4.09	2.17–7.71	<0.001			
Constipation	1.55	0.87–2.76	0.14			
Bullied	1.08	0.51–2.29	0.84			

Depression and anxiety were medically-diagnosed, while constipation, nausea, unhappiness and a history of being bullied were reported by the adolescent at age 17 years. OR = odds ratio; CI = confidence intervals. P values are for associations between self-reported poor-fair overall health rating, and gastrointestinal symptoms, depression, anxiety and unhappiness in adolescents. P < 0.05 is considered statistically significant.

## Discussion

In our study, abdominal pain was common in children and adolescents, particularly females. About one-third of 17-year-old adolescents experienced abdominal pain, lasting at least three hours in one quarter. Abdominal pain was associated with hard stool consistency, nausea and depression in adolescents. Further, adolescents with abdominal pain were more than twice as likely to have a history of depression. Children with frequent or recurrent abdominal pain or painful bowel motions had a higher likelihood of constipation during childhood, and frequent abdominal pain, depression or anxiety during adolescence, compared with other children. Also, feeling unhappy during childhood was prospectively associated with recurrent abdominal pain at age 17 years.

Consistent with previous reports, females reported more frequent abdominal pain than males^9,12,20^ that was associated with a higher prevalence of depression and anxiety^21–24^. The bidirectional relationship between abdominal pain and depression or anxiety in adolescents highlights the risk of progression of one in relation to the other^21^. The improvement in abdominal pain with bowel motions in the majority of adolescents in our study, and the association with depression and anxiety raises the possibility of a functional abdominal pain disorder such as irritable bowel syndrome in some of the adolescents. An association between persistent abdominal pain in children, prevalent poor health and emotional disorders in parents, and increased future risk of adult psychiatric disorders has previously been described in a population-based longitudinal study^25^. Our observation that adolescents living in single parent homes had a higher prevalence of abdominal pain is consistent with a previous report regarding the influence of family composition and functioning on abdominal pain in children^26^.

Co-existing non-organic abdominal complaints, depression, anxiety and fatigue identify individuals at risk of chronicity of symptoms, who may benefit from interventions to ameliorate their symptom complaints and illness behaviour^27^. This is reinforced by our observation that self-reported unhappiness was associated with recurrent abdominal pain independent of a diagnosis of depression or anxiety. This suggests that the emotional burden of abdominal pain may be substantial and under-appreciated in the absence of a formal diagnosis of depression or anxiety. Recurrent abdominal pain could be a source of unhappiness or conversely unhappiness could manifest somatically as abdominal pain. A holistic approach to caring for patients with abdominal pain and/ or emotional concerns is therefore important.

The fact that abdominal pain increased with the frequency of being bullied at school is concerning. Bullying may have considerable effects on emotional wellbeing in adolescents, with anxiety, depression and gastrointestinal symptoms as related somatic consequences. Bullying may not only serve as a direct trigger of emotional difficulties, but may also perpetuate symptoms of distress. Conversely, the adolescent experiencing distress may also be more vulnerable to being bullied by others. Victims of bullying have been shown to experience more psychosomatic symptoms, including abdominal pain, than non-victims^28,29^ and also subsequent adverse health-related quality of life^30^. Thus, abdominal pain in bullied adolescents could be a somatic feature of school anxiety.

Strengths of our study are the large cohort size derived from a well-characterized population-based cohort with comprehensive longitudinal assessments and examined domains including gastrointestinal symptoms, prospectively recorded medications and medical diagnoses. During the 17-year follow-up, the Raine Cohort was considered to be representative of the broader Western Australian population from birth through to adolescence, with characteristics of the families being similar to contemporaneous Western Australian families^19^.

Our study has several limitations, as it is an observational study and with serial assessment there has been cohort attrition. Symptoms relied on recall during each cross-sectional assessment, particularly parent report for children, as is common in clinical practice and many studies on functional gastrointestinal disorders using the Rome criteria^10^. Abdominal pain-related questions applied in the various cross-sectional assessments during childhood and adolescence were different, without standardized diagnostic criteria and relying on parent report for children but self-report by adolescents aged 17 years. A history of anxiety or depression relied on a diagnosis by the primary care physician, reported by the parent and/or adolescent. Also, child unhappiness was reported by parents during childhood ages, with potential for over- or underestimation of symptoms compared with adolescent self-report of unhappiness at ages 14 and 17 years. Consequently, changes in the incidence and types of presumed functional abdominal pain disorders from early childhood into adolescence, and influence of parental anxiety on these could not be determined from the study design. While these factors could potentially increase the risk of bias, the relationship between abdominal pain and unhappiness, depression or anxiety in longitudinal analysis remained consistent during longitudinal assessments. While our study describes associations, we are unable to ascribe causality, account for potential medication side effects that could contribute to abdominal pain or other gastrointestinal symptoms and could not determine whether antidepressants were used for depression, anxiety or adjuncts to analgesia. However, given the stringent guidance on antidepressant medication use in children and adolescents the most likely indications would be depression and anxiety. Since somatic pain at other sites is common in individuals with abdominal pain, use of analgesics cannot be attributed to abdominal pain alone^23^. Associations between abdominal and extra-abdominal somatic pain was beyond the scope of this study. We are also unable to ascertain the direction of association between abdominal pain and other gastrointestinal symptoms, depression, anxiety or bullying from this study, as both abdominal pain and reports of unhappiness were commonly reported. However, chronic or recurrent physical health problems, particularly pain, are known to associate with higher rates and severity of depression and often precede depression^31^.

Other possible explanations for the association between abdominal pain and depression are abdominal pain as an autonomic and somatic symptom of depression, anxiety or somatization disorder^31,32^. Thus, recurrent abdominal pain may be a somatic manifestation of emotional distress, legitimise the experience of distress or represent less stigmatised help-seeking behaviour. For instance, in the Raine Cohort, back and neck pain have also been linked with depression and anxiety in adolescents^33^. Arguably, a number of children and adolescents may find somatic communication of emotional distress more convenient than describing low mood or anxiety at home, school and among peers, and with a more engaging universal response than communicating emotional distress. A previous study concluded that maternal anxiety was the most consistent predictor of subsequent abdominal pain, school absence and anxiety disorders in children^34^. One could hypothesise that somatic complaints may be conditioned in a small proportion of youth. With a high proportion of individuals not receiving a satisfactory explanation for their abdominal pain^9^, there is a risk of chronicity of inadequately treated abdominal pain that may be associated with anxiety, depression and self-medication with analgesics, other drugs or substance use. In our study, adolescents with abdominal pain described themselves as being more unhappy than those without abdominal pain, at both ages 14 and 17 years, suggesting potential chronicity of dysphoria or unhappiness. This description was unrelated to diagnosed depression, highlighting the importance of understanding the significance of, and possibly enquiry regarding, the state of happiness or unhappiness in children and adolescents. Notably, adolescents experiencing abdominal pain were also twice as likely to describe their health as not excellent, compared with those not experiencing abdominal pain. These findings underscore the importance of considering the mental health burden of physical illness and vice versa. Nevertheless, there are inconclusive reports of benefits of antidepressant medications for treatment of recurrent abdominal pain in children and adolescents^35^. Sex-differences in the gut microbiome through the life-course have been associated with mental health disorders, including anxiety and depression^36,37^, as well as with functional gastrointestinal disorders^38^. Consequently, influences of the gut microbiome on the gut-brain axis may contribute to the link between abdominal pain and the mental health of adolescents. Knowledge regarding impacts of the gut microbiome on gut-to-brain and brain-to-gut pathways associated with gastrointestinal and neuropsychiatric phenotypes of functional abdominal pain continue to evolve^39^. In the interim, in the absence of alarm symptoms from history and physical examination, primary care physicians should explain to the parent, adolescent or child that abdominal pain is common and rarely associated with disease^40^.

In conclusion, recurrent abdominal pain is common in children and adolescents and is frequently associated with other gastrointestinal symptoms, and adverse emotional wellbeing, including current or subsequent history of depression, anxiety, unhappiness, being bullied at school, and lower self-perceived health status. Adolescents with abdominal pain demonstrate increased health-seeking behavior, including health professional consultation or medication use, and ill-health. Abdominal pain may also be a somatic feature of underlying emotional distress, masking or delaying psychological help-seeking in children and adolescents. The “duration of unrecognized or untreated distress” may further contribute to chronicity. A comprehensive approach to assessment and management of adolescents suffering from recurrent, persistent or chronic abdominal pain should involve an understanding of the burden of the somatic symptom pattern, psychosocial factors, and family factors on severity and chronicity. Health practitioners have an important role to play in identifying and managing risk associations in adolescents with abdominal pain. Since chronic abdominal pain often precedes depression and chronic use of analgesics, appropriate psychological or pharmacological intervention to manage abdominal pain are critical before a worsening trajectory of depression or anxiety develops in children and adolescents. This temporal relationship is worth further exploration as recurrent and persistent abdominal pain symptoms may be a prodrome of depression and anxiety or vice versa in youth. Future studies should explore not only the “gut - feelings” relationships between abdominal pain, gastrointestinal symptoms, other somatic pain sites and mental health, but also the influence of parent factors and family functioning. Finally, the impact of chronic abdominal pain on parents and siblings of the child or adolescent experiencing abdominal pain should not be overlooked.
